# Trajectories of disease courses in the inception cohort of newly diagnosed patients with JIA (ICON-JIA): the potential of serum biomarkers at baseline

**DOI:** 10.1186/s12969-021-00553-x

**Published:** 2021-05-01

**Authors:** Margarita Ganeva, Sabrina Fuehner, Christoph Kessel, Jens Klotsche, Martina Niewerth, Kirsten Minden, Dirk Foell, Claas H. Hinze, Helmut Wittkowski

**Affiliations:** 1 Department of Pediatric Rheumatology, Medical University Sofia, Sofia, Bulgaria; 2 Department of Pediatric Rheumatology and Immunology, University Hospital Münster, Albert-Schweitzer-Campus 1, Building D3, D-48149 Muenster, Germany; 3 Epidemiology Unit, German Rheumatism Research Center, Berlin, Germany

## Abstract

**Objective:**

Juvenile idiopathic arthritis (JIA) is a heterogeneous group of inflammatory joint disorders with a chronic-remitting disease course. Treat-to-target approaches have been proposed but monitoring disease activity and predicting the response to treatment remains challenging.

**Methods:**

We analyzed biomarkers and their relationship to outcome within the first year after JIA diagnosis in the German Inception Cohort of Newly diagnosed patients with JIA (ICON-JIA). CRP, CXCL9, CXCL10, CXCL11, erythrocyte sedimentation rate, G-CSF, IL-6, IL-17A, IL-18, MCP-1, MIP-1α, MMP-3, S100A8/A9, S100A12, TNFα, and TWEAK were measured at baseline and 3 months later.

**Results:**

Two-hundred-sixty-six JIA patients with active disease at baseline were included, with oligoarthritis and rheumatoid factor-negative polyarthritis representing the most frequent categories (72.9%). Most biomarkers were elevated in JIA compared to healthy pediatric controls. Patients with systemic JIA had higher CRP, S100A8/A9 and S100A12 levels compared to other JIA categories. Baseline levels of TWEAK, G-CSF and IL-18 were lower in oligoarthritis patients with disease extension within 1 year. Increased baseline levels of CRP, S100A8/A9, S100A12 and ESR were associated with the subsequent addition of biologic disease-modifying antirheumatic drugs (DMARDs). Higher baseline ESR, G-CSF, IL-6, IL-17A and TNF levels indicated an increased risk for ongoing disease activity after 12 months.

**Conclusion:**

Our data demonstrate that elevated baseline levels of CRP, S100A8/A9 and S100A12 as well as increased ESR are associated with the necessity to escalate therapy during the first 12 month of follow-up. Furthermore, biomarkers related to Th17 activation may inform on future disease course in previously treatment-naïve JIA patients.

**Supplementary Information:**

The online version contains supplementary material available at 10.1186/s12969-021-00553-x.

## Introduction

Juvenile idiopathic arthritis (JIA) is a heterogeneous group of disorders currently classified according to the International League of Associations for Rheumatology (ILAR) which collectively represent the most common rheumatic diseases in childhood [1]. Despite the progress in JIA therapy, even in contemporary cohorts fewer than half of patients achieve long-term remission [2]. Periodic assessment of the disease activity is needed to allow targeted therapy [3]. Several composite scores/criteria have been developed, containing both clinical and laboratory parameters [4–6].

Biomarkers have shown potential as diagnostic and prognostic tools in JIA, as tools for assessment of disease activity and severity, for determination of the likelihood of clinical remission or relapse, and the response to therapy. Assessment of disease activity in JIA usually involves laboratory markers of inflammation already proven in clinical practice such as erythrocyte sedimentation rate (ESR) and C-reactive protein (CRP). Significant efforts are focused on detecting other serum biomarkers of disease activity that are more sensitive and reliable. Several studies have addressed biomarkers in JIA patients so far, and several candidates were identified in exploratory studies, including many different chemokines and cytokines [7, 8]. For most of these biomarkers, validation studies have not yet been performed. However, serum S100 proteins, including S100A8/A9 (also known as MRP8/14 or calprotectin) and S100A12 are extraordinarily elevated in patients with active systemic JIA, may indicate subclinical inflammation, and may identify patients with non-systemic JIA with a good response to tumor necrosis factor alpha (TNFα) blockade [9–12]. Biomarkers can ideally be implemented in routine clinical care to identify JIA patients at particular risk for a complicated disease course, e.g., for not achieving disease inactivity, a relapse of the disease or the occurrence of complications. Previously, ESR and serum S100A12 were identified as biomarkers for a higher risk of subsequent development of JIA-associated uveitis in the prospective Inception Cohort of Newly diagnosed patients with JIA (ICON-JIA) study [13].

The objective of this study was to describe the association of baseline serum biomarkers and inflammatory parameters with the 12-month outcome of active JIA patients within the ICON-JIA study. Specifically, we were seeking biomarkers (1) indicating the risk of disease extension within oligoarthritis patients, (2) predicting the need of treatment escalation with subsequent use of biological DMARDs, and (3) predicting attainment of inactive disease in treatment-naïve patients.

## Materials and methods

### Patients

Patients less than 16 years of age with recently diagnosed JIA (less than 12 months before inclusion) were enrolled in ICON-JIA, an ongoing national prospective observational, multicenter study which started in 2010. This inception cohort collects real-life data, i.e. patients are treated based on the preferences of their individual providers, and there were no standardized treatment protocols. Clinical and laboratory parameters were recorded quarterly during the first year and semiannually thereafter. Of the 954 enrolled patients, 266 patients were selected for this analysis based on availability of serum samples at baseline and 3-month follow-up (261 out of 266 patients had serum samples at both time points) as well as active disease at baseline. The latter was defined as clinical Juvenile Arthritis Disease Activity Score (cJADAS)10 ≥ 1.1. For control purposes, blood samples were also taken from 16 children with non-inflammatory conditions. The study was approved by the ethics committees of the University of Muenster (reference numbers 2010–267-b-S and 2015–670-f-S) and the Charité University Medicine Berlin (reference number EA1/056/10). All parents and patients (of 8 years and above) gave their informed consent at study inclusion according to the Declaration of Helsinki.

### Disease activity

JIA disease activity states were assessed at inclusion and at the 3- and 12-months follow-up visits. Disease activity was measured with the clinical JADAS10 (cJADAS10) with a range of 0 to 30 (lower is better). The cJADAS10 comprises 3 variables: physician global rating on a 21-point numeric rating scale (NRS) from 0 to 10 (0 = no disease activity; 10 = maximal disease activity); parent or child global rating on a 21-point NRS from 0 to 10 (0 = no disease activity; 10 = maximal disease activity); active joint count from 0 to 10. We defined disease activity categories for all patients on an ordinal scale, based on previously defined cJADAS10 cut-offs for patients with oligoarthritis or polyarthritis (inactive disease: ≤1 in both categories; low disease activity: 1.1–1.5 or 1.1–2,5; moderate disease activity: 1.51–4 or 2.51–8.5; high disease activity: > 4 or > 8.5, respectively) [14]. Children with JIA in ILAR categories other than oligo- or polyarthritis were categorized based on the number of joints affected during the disease course (≤4 or > 4, respectively). All the 266 patients included in the present study had active disease at baseline (cJADAS10 ≥ 1.1). In 6 out of 266 patients, information on disease activity at 3-month was not available. For all the 266 patients included in the study information on disease activity at the 12-month follow up was present.

### Biomarker analyses

Blood samples collected at baseline and at the 3-month follow-up visit were tested for CRP, S100A8/A9, and S100A12 as well as ESR. Concentrations of CXCL9, CXCL10, CCL11 (Eotaxin), G-CSF (granulocyte colony stimulating factor), interleukin (IL)-6, IL-17A, IL-18, MCP-1 (monocyte chemoattractant protein 1), MIP-1α (macrophage inflammatory protein-1α), MMP-3 (matrix metalloproteinase-3), TNFα (tumor necrosis factor alpha) and TWEAK (tumor necrosis factor-like weak inducer of apoptosis) were quantified according to respective manufacturer instructions using bead array assay reagents purchased from Bio-Techne (Minneapolis, MN, USA) and Thermo Fisher Scientific (ProcartaPlex; Waltham, MA, USA). The choice of biomarkers was based on prior studies [7]. Data acquisition was performed on a MAGPIX Instrument (Merck, Darmstadt, Germany) using xPONENT v4.2 software (Luminex, Austin, TX, USA). Data were analyzed by ProcartaPlex Analyst Software (v1.0, Thermo Fisher Scientific). In 261 out of 266 patients serum samples were available at both time points (baseline and 3 month follow up).

### Outcome

The primary outcome of the study was the association between baseline biomarker levels, patient characteristics, disease activity and extension as well as subsequent escalation of therapy.

### Statistical analyses

Statistical analyses were performed using GraphPad Prism software (v7.05 for Windows, v8.0 MacOSX; GraphPad Software, La Jolla, California, USA). Descriptive statistics were calculated for all variables and are presented as absolute frequencies, as median values, range and interquartile range (IQR). Since the biomarker values were not normally distributed, non-parametric statistical testing was performed for the comparison of the different subgroups defined. Statistical test-methods are indicated in the figure legends. Differences between groups were considered to be significant at a *P* value of < 0.05. Distributions were compared via chi square test. Receiver operating characteristic curve (ROC) analyses were performed to assess the predictive accuracy of serum biomarkers and optimal cut-off levels to predict remission were defined. A heatmap of all quantified baseline parameters based on average linkage and Spearman rank correlation was generated using Heatmapper [15].

## Results

### Clinical characteristics and disease course during the study

**Fig. 1:**
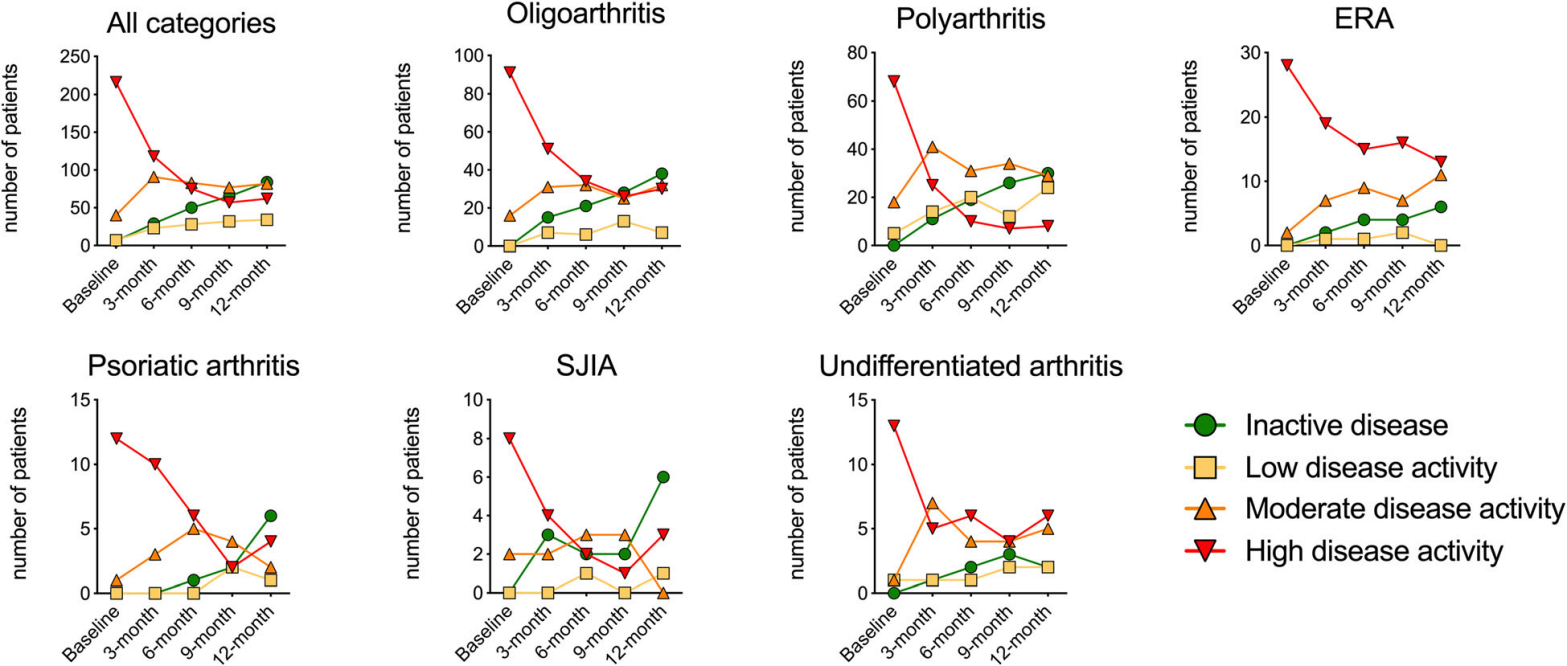
Disease activity course across different JIA categories. Disease activity was assessed at baseline and 3-month intervals thereafter via the clinical juvenile arthritis disease activity score (cJADAS) and converted to an ordinal scale via validated cut-offs that were established for patients with oligoarthritis and polyarthritis. Children with JIA in ILAR categories other than oligo- or polyarthritis were categorized based on the number of joints affected during the disease course (≤4 or > 4, respectively). Low disease activity: 1.1–1.5 in oligoarthritis or 1.1–2.5 in polyarthritis; moderate disease activity: 1.51–4 or 2.51–8.5; high disease activity: > 4 or > 8.5, respectively

Baseline characteristics of all 266 patients are presented in Table 1. The most frequent JIA categories observed were oligoarthritis in 107 (40.2%), rheumatoid factor negative (RF-) polyarthritis in 87 (32.7%), enthesitis-related arthritis (ERA) in 30 (11.3%), and undifferentiated arthritis in 15 (5.6%), with other categories each representing less than 5% of patients. At the 12-months visit, 88 of 266 (33.1%) had inactive disease (cJADAS≤1). The highest proportion of inactive disease was achieved in the systemic arthritis category with 6 out of 10 (60%), and the lowest in the undifferentiated category with 2 out of 15 (13.3%) and enthesitis-related arthritis (ERA) with 6 out of 30 (20.0%) patients. In 43 out of 229 patients sustained inactive disease from the 9-month to the 12-month visit was observed; data were missing for 37 patients at the 9-month visit. At the 12-month visit 34 (12.8%), 80 (30.1%), and 64 (24.1%) had low, moderate, and high disease activity, respectively (Fig. 1). Sex, age at diagnosis and ANA positivity did not correlate with active versus inactive disease state at the 12-month follow-up. Furthermore, when considering the active joint count (AJC) at the 12-month follow up visit, 166 out of 266 (62.4%) patients had achieved an AJC of zero (Supplementary Table 1).

**Table 1:** Baseline characteristics of study cohort

	Oligoarthritis	RF- polyarthritis	RF+ polyarthritis	ERA	PsA	systemic JIA	Undifferentiated arthritis
Number of patients (% of all patients)	107/266 (40.2%)	87/266 (32.7%)	4/266 (1.5%)	30/266 (11.3%)	13/266 (4.9%)	10/266 (3.8%)	15/266 (5.6%)
Number of girls (% within category)	82/107 (76.6%)	70/87 (80.4%)	3/4 (75%)	8/30 (26.6%)	9/13 (69.2%)	4/10 (40.0%)	10/15 (66.6%)
Age in years at diagnosis, median (range)	5.0 (0.5–15.6)	6.1 (1.0–16.2)	10.9 (8.5–12.0)	10.8 (1.4–16.1)	10.2 (1.4–15.7)	5.9 (0.9–13.4)	8.1 (3.0–14.6)
Duration of symptoms in months, median (range)	8.2 (0.0–57.1)	10.4 (0.6–139.6)	6.5 (1.0–9.7)	12.5 (1.2–70.1)	7.8 (2.2–14.0)	5.2 (1.4–10.7)	10.1 (0.4–31.2)
Duration between diagnosis and enrollment in days, median (range)	42 (0–327)	28 (0–363)	110 (1–142)	76 (0–363)	62 (6–232)	72 (7–327)	42 (3–261)
cJADAS-10, median (range)	8.45 (2–18.5)	13.7 (1.5–27)	8.62 (4–14.5)	10.45 (2.5–22)	14.3 (4–23.5)	14.25 (2–24.5)	11.5 (1.5–22.5)
Active joint count, median (range)	1.8 (0–7)*	9.9 (0–57)	3 (2–5)	3.6 (0–16)	8.2 (0–29)	11.4 (0–57)	5.9 (1–31)
CRP in mg/l, median (range)	6.53 (0–100)	10.8 (0–124)	0.57 (0–1.1)	9.56 (0–65)	4.8 (0–15)	47.87 (4.7–96.8)	17.2 (0–53)
Number of patients with previous medications (% within category)							
Synthetic DMARDs	54/107 (50.5%)	65/87 (74.7%)	3/4 (75%)	14/30 (46.7%)	8/13 (61.5%)	7/10 (70%)	5/15 (33.3%)
Systemic glucocorticoids, current	19/107 (17.8%)	41/87 (47.1%)	1/4 (25%)	6/30 (20%)	7/13 (53.8%)	10/10 (100%)	3/15 (20%)
Systemic glucocorticoids, last 6 months before baseline	20/107 (18.7%)	27/87 (31.03%)	1/4 (25%)	7/30 (23.4%)	6/13 (46.1%)	7/10 (70%)	4/15 (26.7%)
Intraarticular glucocorticoids, last 6 months before baseline	62/107 (57.9%)	47/87 (54.02%)	1/4 (25%)	8/30 (26.7%)	3/13 (23.1%)	3/10 (30%)	6/15 (40%)
Biologic DMARDs	2/107 (1.9%) ADA - 2	2/87 (2.3%) ETN - 2	0 (0.0%)	3/30 (10%) ETN - 3	1/13 (7.7%) ETN - 1	3/10 (30.0%) ANA – 1 CAN – 1 TCZ – 1	1/15 (6.7%) ETN - 1
Treatment naive	27/107 (25.2%)	7/87 (8.0%)		12/30 (40.0%)	3/13 (23.1%)		5/15 (33.3%)

Abbreviations: *ADA* adalimumab; *ANA* anakinra; *CAN* canakinumab; *CRP* C-reactive protein; *DMARD* disease-modifying antirheumatic drug; *ERA* enthesitis-related arthritis; *ETN* etanercept; *JADAS* juvenile arthritis disease activity score; *PsA* psoriatic arthritis; *RF* rheumatoid factor; *TCZ* tocilizumab

*4 patients had already developed extended oligoarthritis (≥5 affected joints) at baseline: 3 patients had 5 active joints, 1 patient had 7 active joints

In the whole cohort 212 of the patients were not treatment naïve at baseline with 156 of those being treated with synthetic disease-modifying anti-rheumatic drugs (DMARDs), 130 of the 212 patients have received intraarticular corticosteroid injections in the last 6 months before inclusion in the study and 87 of the patients who were not treatment naïve were taking systemic glucocorticoids at time of inclusion with 12 patients already being treated with biological DMARDs at time of enrollment in the study (Table 1).

### Variation in biomarkers at baseline

**Fig. 2:**
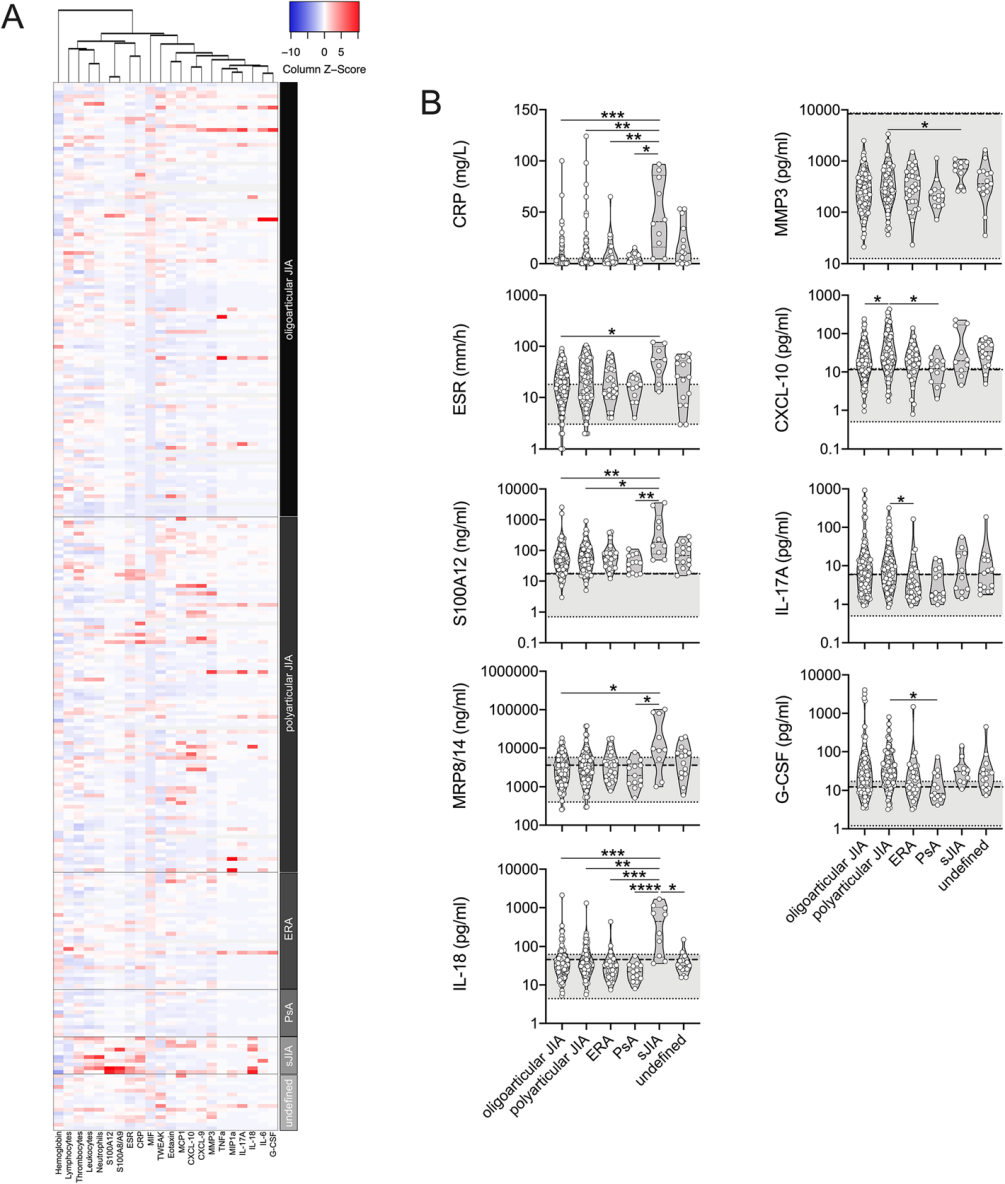
Serum biomarkers and inflammatory parameters at study inclusion. **a**, **b** Clinical laboratory and inflammatory parameters of JIA patients (*n* = 266) were assessed at participating clinical centers upon study inclusion (baseline). Corresponding serum analyte levels were quantified by multiplexed bead array assay as well as ELISA (S100A8/A9, S100A12). **b** Parameters with significant differences are shown in relation to healthy control levels (range: light grey shading, 95th percentile: broken line). Data are presented as violin plots with individual values and were analyzed by Kruskal Wallis followed by Dunn’s multiple comparison test. * = *p* < 0.05, ** = *p* < 0.01, *** = *p* < 0.001, **** = *p* < 0.0001

Biomarker levels at baseline were highly variable between patients across and within JIA categories (Fig. 2a). Several biomarkers, including CRP, ESR, IL-18, S100A8/A9 and S100A12 were elevated in systemic JIA when compared to other JIA categories, whereas inter-category differences in CXCL10, G-CSF, IL-17A and MMP-3 levels were not consistent (Fig. 2b). Other biomarkers did not demonstrate inter-category differences at baseline (Supplementary Figure 1). Most serum biomarkers, across all JIA categories, were substantially higher when compared to levels measured in healthy controls (Fig. 2b).

### Biomarkers in active compared to inactive disease

In order to assess whether the biomarkers could discriminate active disease (cJADAS≥1.1) from inactive disease (cJADAS≤1), biomarker levels were compared between patients with active disease (*n* = 228) and those with inactive disease (*n* = 32) at the 3-month visit. Levels were elevated in active disease for ESR (median 12 [range 7–19] v. 6 [4–12.5] mm/h, *p* < 0.001 and S100A12 (37 [21–64] v. 19 [12–37] ng/ml, *p* = 0.003) and these differences were mostly driven by patients with moderate or high disease activity (Supplementary Figure 2).

### Prediction of disease extension in patients with an oligoarthritis baseline diagnosis

**Fig. 3:**
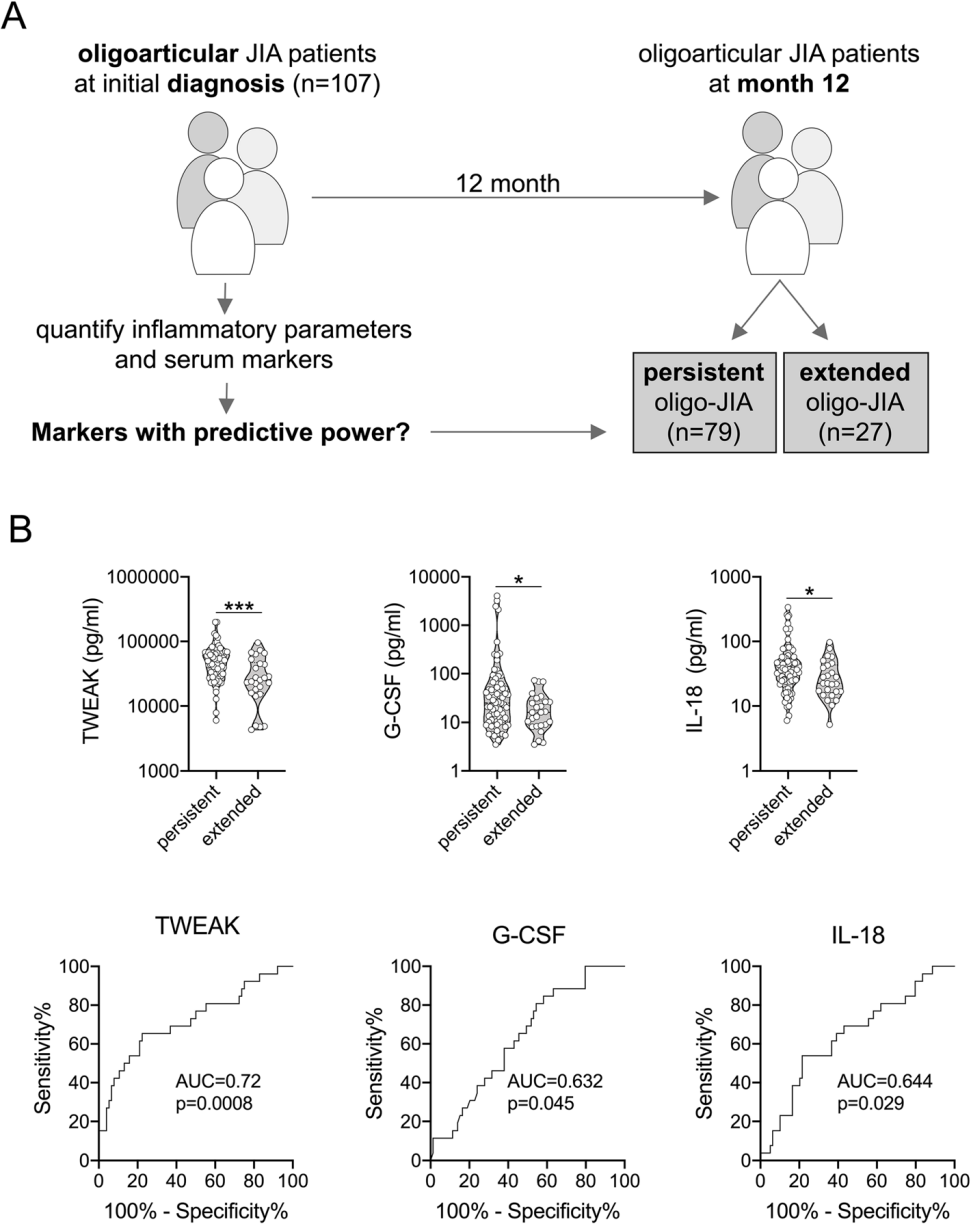
Association of baseline serum biomarkers with disease extension at 12 months. **a** Inflammatory parameters and serum biomarkers of patients diagnosed as oligoarticular JIA at study inclusion (*n* = 107) were associated with clinical disease (persistent or extended oligoarthritis) according to ILAR at 12-month follow-up. **b**, **c** Indicated serum biomarkers revealed significantly different levels at baseline when associated with clinical outcome at 12-month follow-up. Data are presented as violin plots with individual values or ROC and were analyzed by Mann-Whitney U test. * = *p* < 0.05, *** = *p* < 0.001

Of the 107 patients with oligoarthritis at baseline, 27 (25.5%) were categorized as extended oligoarthritis and 79 (74.5%) as persistent oligoarthritis (Fig. 3a) when re-assessed at the 12-month follow-up visit. One patient with an initial oligoarthritis diagnosis was later categorized as having PsA and therefore excluded from the following analysis. G-CSF, IL-18 and TWEAK serum levels at baseline were higher in patients with persistent oligoarthritis at 12 months (baseline median of G-CSF 24.91 pg/ml; IL-18 35.93 pg/ml; TWEAK 45289 pg/ml) when compared to patients with extended oligoarthritis (baseline median of G-CSF 15.93 pg/ml; IL-18 22.41 pg/ml; TWEAK 23988 pg/ml) at 12 months, with modest accuracy (Fig. 3b). However, the patients had received different treatment modalities prior to or at baseline: systemic glucocorticoids were received prior to biomarker measurement by 14 out of 79 (17.7%) patients who still had persistent oligoarthritis at the 12-month follow-up, and by 12 out of 27 (44.4%) with extended oligoarthritis by 12 months of observation (*p* < 0.01, chi square). Intraarticular glucocorticoids were received by 44 out of 79 (55.7%) with persistent oligoarthritis, and by 17 out of 27 (63.0%) with extended oligoarthritis (*p* = 0.50, chi square). Synthetic DMARDs were received by 33 out of 79 (41.8%) with persistent oligoarthritis, and by 18 out of 27 (66.7%) with extended oligoarthritis (*p* = 0.03, chi square).

### Association of baseline biomarkers with need for treatment escalation

**Fig. 4:**
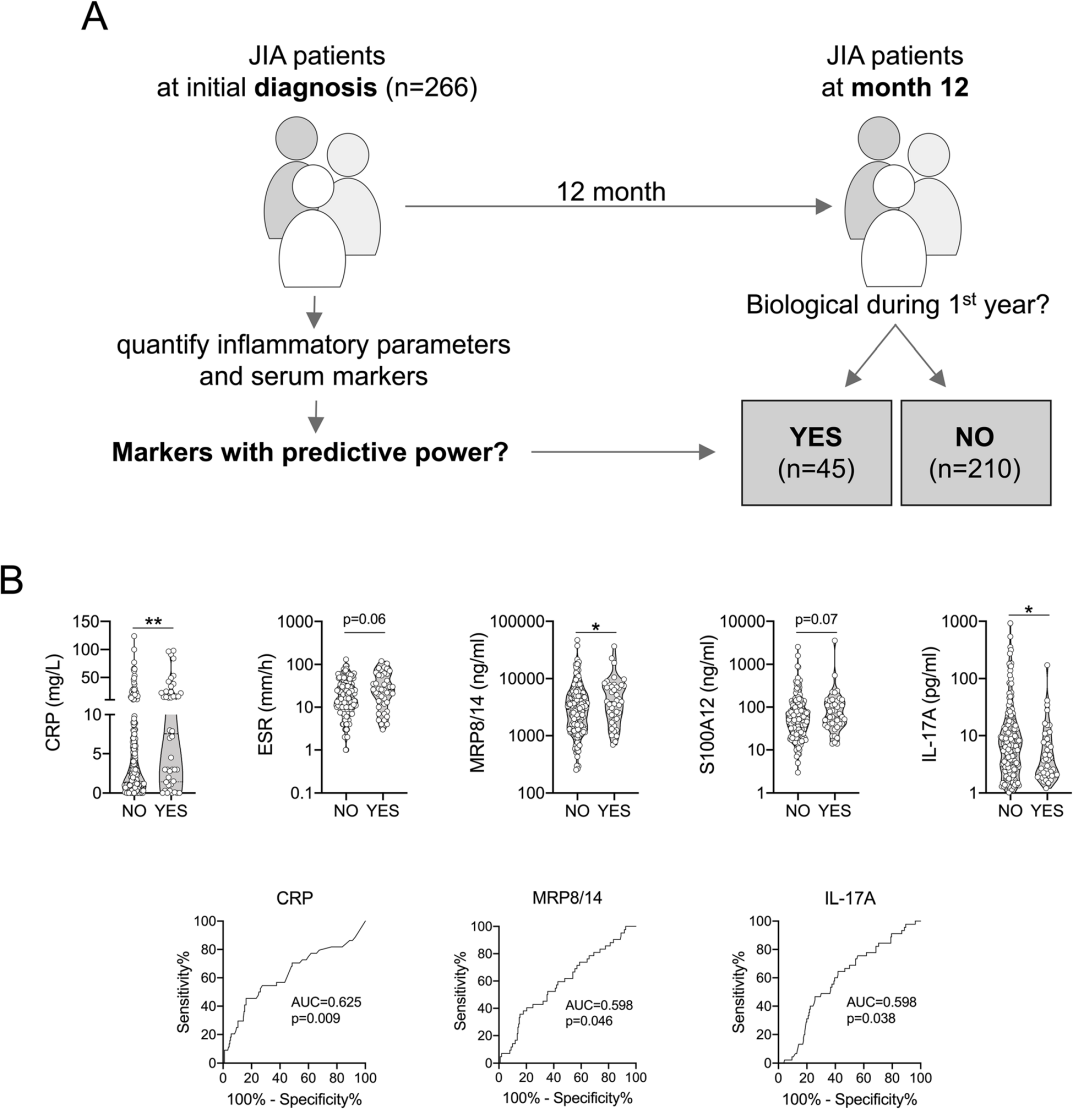
Association of baseline serum biomarkers and inflammatory parameters with treatment escalation during the first 12 months. **a** Inflammatory parameters and serum biomarkers of JIA patients at study inclusion (*n* = 266) were analyzed according to whether or whether not receiving biological DMARDs during the first year. **b**, **c** Indicated parameters assessed at baseline performed (almost) significantly different when associated with eventual treatment escalation up to 12-month follow-up. Data are presented as violin plots with individual values or ROC (only significantly different parameters) and were analyzed by Mann-Whitney U test. * = *p* < 0.05, ** = *p* < 0.01

In the whole cohort, 45 of 266 (16.9%) patients (8 oligoarthritis, 18 RF- polyarthritis, 1 RF+ polyarthritis, 5 ERA, 4 PsA, 5 systemic JIA, 4 undifferentiated arthritis) required addition of a biological DMARD after a median of 7.0 months (IQR 4.0–9.0 months) (Fig. 4a). The JIA categories with the highest proportion of biological DMARD treatment were systemic JIA (5 of 10 [50%]), PsA (4 of 13 [31%]) and polyarthritis (19 of 91 [21%]). The biological DMARDs used were TNF blockers (41 of 45 [91.1%]), anakinra or canakinumab (3 of 45 [6.7%]) and tocilizumab (1 of 45 [2.2%]). Forty-one of 45 (91.1%) had previously received methotrexate. Several biomarkers measured at baseline were associated with the addition of biological DMARDs (Fig. 4b). Higher CRP, S100A8/A9, and S100A12 levels and higher ESR, and lower IL-17A at baseline were associated with the need of subsequent addition of biological DMARDs. This analysis was repeated after excluding patients with systemic JIA from the analysis, showing similar results.

### Prediction of clinically inactive disease at 12 months by baseline biomarker levels

**Fig. 5:**
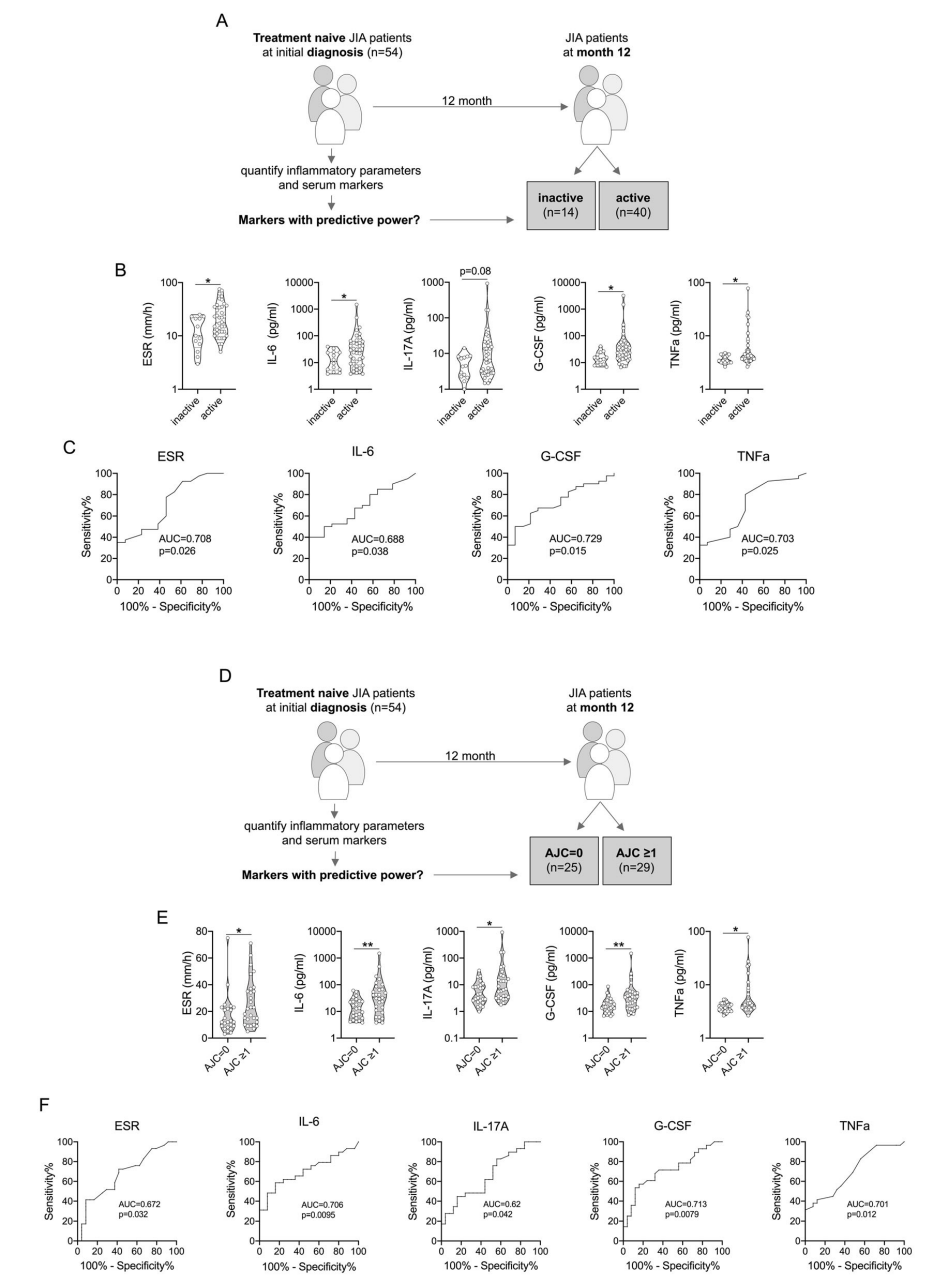
Association of baseline serum biomarkers and inflammatory parameters with disease activity outcome at 12 months. **a** Inflammatory parameters and serum biomarkers of treatment naïve patients at study inclusion (*n* = 54) were associated with clinical disease activity according to cJADAS-10 at 12-month follow-up (inactive disease: ≤1). **b**, **c** Indicated parameters assessed at baseline were (almost) significantly different when associated with clinical outcome at 12-month follow-up. **d** In the same sub-cohort of treatment naïve patients (*n* = 54), baseline inflammatory parameters and serum biomarkers were associated with clinical disease activity according to active joint count (AJC) at 12-month follow-up. **e**, **f** Indicated parameters assessed at baseline revealed significant differences when associating with clinical outcome at 12-month follow-up. Data are presented as violin plots with individual values or ROC (only significantly different parameters) and were analyzed by Mann-Whitney U test. * = *p* < 0.05, ** = *p* < 0.01

Fifty-four patients (27 oligoarthritis, 7 RF- polyarthritis, 12 ERA, 3 psoriatic arthritis (PsA) and 5 undifferentiated) had not received DMARDs or glucocorticoids at baseline and, thus, were treatment-naive. According to cJADAS criteria of these 54 patients, 14 (25.9%) had inactive disease (cJADAS≤1) and 40 (74.1%) had active disease (cJADAS≥1.1) at the 12-month follow-up visit. Furthermore, 25 out of 54 (46.3%) patients achieved an AJC of zero at the 12-month visit. Several biomarkers measured at baseline were significantly associated with these outcomes (Fig. 5). Higher ESR as well as G-CSF, IL-6, and TNFα levels at baseline were associated with active disease at 12 months (Fig. 5b), however, with only modest accuracy, based on an area-under-the-curve (AUC) ranging from 0.69 to 0.73 in ROC curve analysis (Fig. 5c). Similarly, higher ESR as well as serum titers of G-CSF, IL-6, IL-17A, and TNFα were associated with an AJC ≥1 at 12 months (Fig. 5e), again with modest accuracy (AUC in ROC curve analysis ranging from 0.62–0.71) (Fig. 5f). None of the remaining biomarkers measured at baseline demonstrated a significant association with these outcomes at 12 months (Supplementary Figure 3).

## Discussion

Previous reports have already analyzed biomarkers in JIA, although in smaller cohorts [7, 8, 16–19]. A pioneering study in this respect used a broad multiplex approach to analyze 30 cytokines in 65 JIA patients with a median disease duration of 4.4 (0.2–14.0) years of whom 41 had active disease [7]. Serum markers corresponding with JIA disease activity as identified by this study (predominantly CCL2 (MCP-1), CCL3 (MIP-1a), CCL11 (Eotaxin), MIF, CXCL9, CXCL10 and IL-18) strongly supported the biomarker panel design for our analyses.

Even though JIA serum marker analyses have been previously performed, our study followed an advanced approach: a) patients were assessed early in the disease course (recent JIA diagnosis - less than 12 months before inclusion in the study), partially (54 out of 266 patients were treatment naïve) prior to initiation of any therapy, b) bio-samples were routinely collected, c) treatment approaches represented real-life management of the disease, which means no standardized protocol, but the treatment decisions by an experienced pediatric rheumatologist, and d) patients were followed prospectively with close monitoring via validated clinical tools. In part similar, the Canadian ReACCh-Out inception cohort was comparable in scope but did not collect bio-samples and was completed in the early biologic era [20].

The observed variability of numerous biomarkers is not surprising as JIA is both phenotypically and genotypically a very heterogenous disease [7, 21, 22]. Some biomarkers revealed differences between JIA categories. For example, the tendency for higher serum IL-17A in the oligoarthritis category is consistent with previous findings in peripheral blood and also in synovial tissue [7, 23, 24]. As expected, patients with systemic JIA had substantially elevated S100A8/A9, S100A12 and CRP levels compared to patients with other JIA categories [11, 12, 25].

The primary question of our study was whether baseline quantification of serum biomarkers and inflammatory parameters may inform about a) disease extension, b) escalation of therapy within a 12-month follow-up, and c) the likelihood to attain a status of inactive disease (cJADAS≤1) after 1 year. We first analyzed baseline biomarker data regarding their potential in predicting the development of extended oligoarthritis compared to persistent oligoarthritis. Here we found TWEAK, G-CSF and IL-18 levels to significantly associate with subsequent extended oligoarthritis. Strikingly, baseline cytokine levels were higher in patients with subsequent persistent oligoarthritis, even though extended oligoarthritis would be expected to demonstrate more inflammatory activity [26, 27]. A possible explanation is that patients with subsequent extended oligoarthritis had received systemic glucocorticoids in a higher proportion prior to or at the time of sampling blood, which may suppress the concentrations of proinflammatory mediators. We can only speculate that G-CSF, IL-18 and TWEAK serum levels may have been impacted to a larger degree by glucocorticoids compared to the other biomarkers tested.

Secondly, we demonstrated higher baseline ESR, CRP and S100A8/A9 levels in patients in whom bDMARDs were subsequently added to the respective treatment regimen. Hence, ESR, CRP and S100A8/A9 may serve as biomarkers for patients with a higher probability to require antirheumatic therapy beyond MTX. This finding is in contrast with previously published results by Moncrieffe et al. who report higher S100A8/A9 and CRP values in a subgroup with better response to MTX [28]. However, the outcomes considered are different between these studies. Furthermore, Moncrieffe et al. collected blood samples prior to initiation of MTX treatment. In our study, all 266 investigated patients - apart from just 54 DMARD-naïve ones - were already under treatment at the time of enrollment in the study. Moreover, the number of patients who required systemic glucocorticoids prior to or at time of inclusion in our study cohort was higher in the group with treatment escalation compared to the group with no escalation of therapy (71.1% vs 31.9%). The latter suggests higher disease activity and hence higher baseline biomarker values in patients in whom bDMARDs will be added. In the present study we included 10 patients with systemic JIA and 5 of them started bDMARD during the first year of follow-up, which composes 11% (5/45) of the patients in this subgroup. Indeed, the systemic JIA subtype is the one with highest median baseline values of S100A8/A9 and CRP compared to all other JIA categories. However, removal of systemic JIA from the data set does not alter the respective sub-analysis results (data not shown). Among the 45 patients in whom bDMARDs were subsequently added, 8 were patients with oligoarthritis. Although bDMARDs are not a part of the official German treatment guidelines for oligoarthritis, they are frequently used in oligoarthritis with refractory disease course or in case of JIA-associated uveitis [29, 30].

Finally, the most ambitious aim was to test whether baseline serum biomarkers would predict the likelihood to attain a status of clinically inactive disease after 1 year. We found that several biomarkers were able to early on inform about trajectories of disease activity outcome, albeit with modest accuracy. In DMARDs-naïve patients who present with higher disease activity and/or AJC at 12-month follow-up, increased levels of IL-6, IL-17A, TNFα and G-CSF at baseline may be indicative of early enhanced Th17 activity. IL-6 is a crucial cytokine promoting Th17 cell differentiation while inhibiting the induction of regulatory T (Treg) cells [29, 30]. Once fully differentiated, Th17 cells by themselves can express TNFα, which in turn promotes pro-inflammatory activation of antigen presenting cells that are required for further T cell activation [31, 32]. IL-17-mediated activation of target cells such as neutrophils results in G-CSF expression, which can promote cell activation and further recruitment to sites of inflammation [33]. Presence and activity of Th17 cells in JIA pathophysiology is well documented and has been observed particularly in oligoarticular JIA [23]. Of note, in our treatment-naïve cohort half of the patients (27/54) had oligoarticular involvement.

The definition that we have used for inactive disease (cJADAS≤1) is rather strict. At the 12-month visit, 88 of 266 (33.1%) patients had inactive disease (cJADAS≤1). However, when active joint count (AJC) is used as a marker of disease activity, 166 of 266 (62.4%) patients had an AJC of zero at the 12-month visit. Nevertheless, this proportion is well in line with previously published data. For example, in the Canadian ReACCh-Out cohort, or in the British CAPS-cohort proportions of inactive disease are comparable or lower [20, 34].

However, we should mention some limitations of the present study, which are a) a small patient number in the treatment-naïve cohort (*n* = 54); b) heterogeneity of different disease categories in regard to treatment duration and treatment regimens at baseline and; c) the absence of a validation cohort. Additionally, there was no standardized treatment protocol applied.

In summary, among patients with active JIA in a real-world scenario, a higher degree of inflammation early in the disease course appears to be associated with persistent and more severe disease in the long-term. This observation may support the theory underlying the “window-of-opportunity” hypothesis, i.e. that the presence of extended inflammation may translate into long-term immunological changes affecting the disease course. However, while this study offers some observational evidence in this regard, further mechanistic studies are needed to outline the evolution of immunological disturbances in patients with JIA.

## Supplementary Information

**Additional file 1.**

## Data Availability

The datasets used and/or analysed during the current study are available from the corresponding author on reasonable request.
