# Supplementary material for: Trajectories of disease courses in the inception cohort of newly diagnosed patients with JIA (ICON-JIA): the potential of serum biomarkers at baseline
Source: Pediatr Rheumatol Online J. 2021 May 1;19:64. doi: 10.1186/s12969-021-00553-x (PMC8088653; doi:10.1186/s12969-021-00553-x)
Supplement: Supplementary file 1 — Additional file 1. [file 12969_2021_553_MOESM1_ESM.docx]

SUPPLEMENTARY MATERIAL

**Trajectories of disease courses on the Inception Cohort of Newly diagnosed patients with JIA (ICON-JIA): The potential of serum biomarkers at baseline**

**Authors:**

Margarita Ganeva^1,2^, Sabrina Fuehner^2^, Christoph Kessel^2^, Jens Klotsche^3^, Martina Niewerth^3^, Kirsten Minden^3^, Dirk Foell^2^, Claas H Hinze^2^, Helmut Wittkowski^2^

**Affiliations:**

^1^Department of Pediatric Rheumatology, Medical University Sofia, Bulgaria; ^2^Department of Pediatric Rheumatology and Immunology, University Hospital Münster, Münster, Germany; ^3^Epidemiology Unit, German Rheumatism Research Center, Berlin, Germany

|  | cJADAS ≤ 1 | AJC=0 | Physician´s Global Assessment  = 0 | Physician´s Global Assessment  ≤1 | Parental NRS  = 0 | Parental NRS  ≤1 | Patient NRS  = 0 | Patient NRS  ≤1 |
| --- | --- | --- | --- | --- | --- | --- | --- | --- |
| 3^rd^ month | 32/260  (12.3%)  N/A n=6 | 107/264  (40.5%)  N/A n=2 | 44/264  (16.7%)  N/A n=2 | 119/264  (45.1%)  N/A n=2 | 38/261  (14.5%)  N/A n=5 | 92/261  (35.2%)  N/A n=5 | 24/106  (22.6%)  N/A n=160 | 48/106  (45.3%)  N/A n=160 |
| 6^th^ month | 49/235  (20.8%)  N/A =31 | 136/239  (56.9%)  N/A n=27 | 55/239  (23.01%)  N/A n=27 | 140/239  (58.6%)  N/A n=27 | 47/254  (18.5%)  N/A n=12 | 110/254  (43.3%)  N/A =12 | 26/107  (24.3%)  N/A n=159 | 59/107  (55.14%)  N/A n=159 |
| 9^th^ month | 65/229  (28.4%)  N/A =37 | 127/234  (54.3%)  N/A n=32 | 66/234  (28.2%)  N/A n=32 | 160/234  (68.4%)  N/A n=32 | 54/253  (21.3%)  N/A n=13 | 141/253  (55.7%)  N/A =13 | 30/110  (27.3%)  N/A n=156 | 63/110  (57.3%)  N/A n=156 |
| 12^th^ month | 88/266  (33.1%)  N/A n=0 | 166/266  (62.4%)  N/A n=0 | 90/266  (33.8%)  N/A n=0 | 198/266  (74.4%)  N/A n=0 | 70/266  (26.3%)  N/A n=0 | 144/266  (54.1%)  N/A n=0 | 34/117  (29.05%)  N/A n=149 | 65/117  (55.5%)  N/A n=149 |
| AJC, active joint count; cJADAS, clinical juvenile arthritis disease activity score; N/A n, number of patients for whom data point not available; NRS, 21-point numerical rating scale with a range of 0-10 | | | | | | | | |

**Table S1. Proportion of inactive disease according to follow up.**

Figure S1. Serum biomarkers and inflammatory parameters at study inclusion. Parameters with non-significant differences (related to Figure 2) are shown in relation to healthy control levels (range: light grey shading, 95^th^ percentile: broken line). Data are presented as violin plots with individual values and were analyzed by Kruskal Wallis followed by Dunn’s multiple comparison test.

**
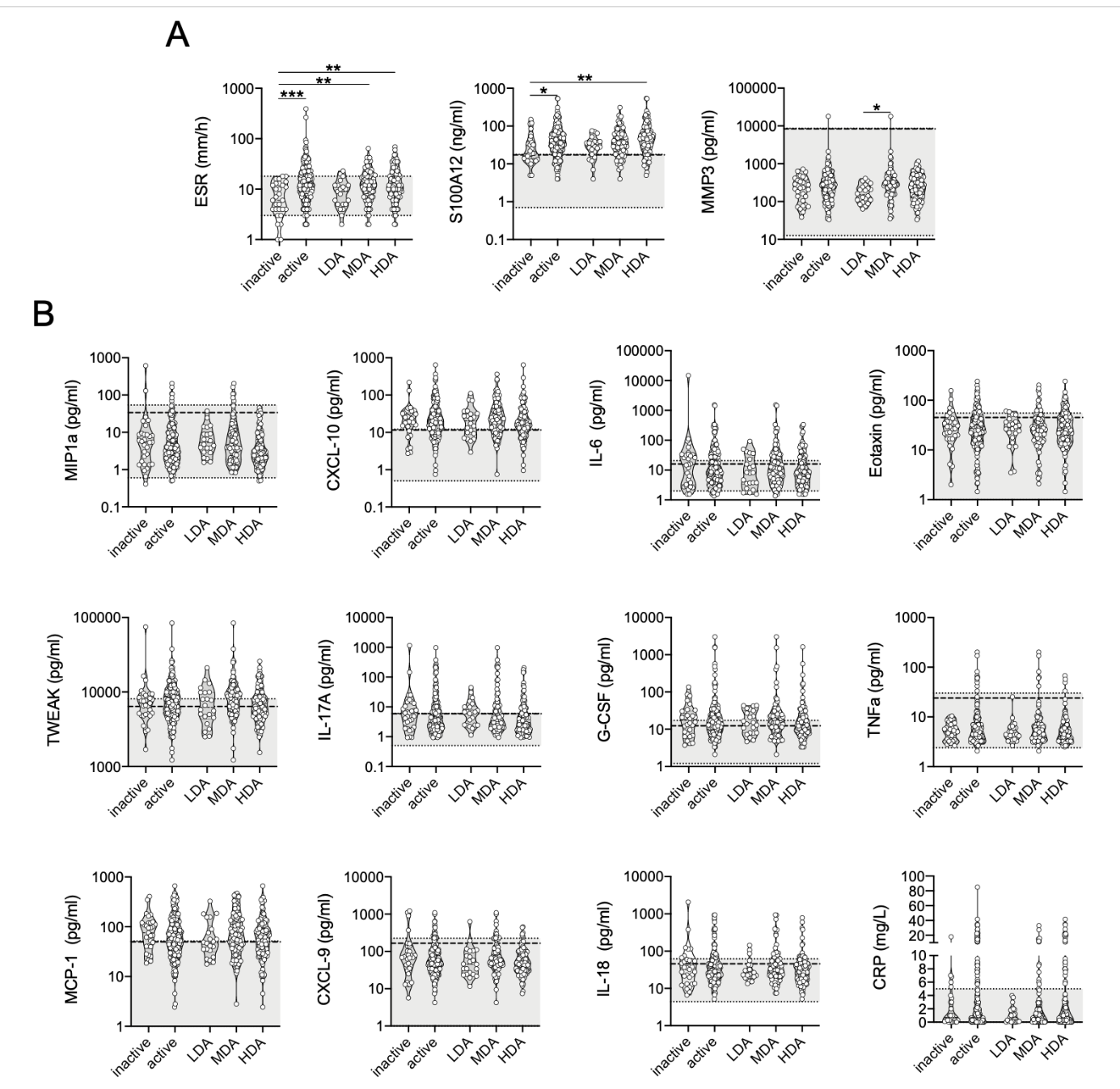
**

Figure S2. Serum biomarkers and inflammatory parameters at three-month follow-up. (A, B) Clinical laboratory and inflammatory parameters of JIA patients (n=266) were assessed at participating clinical centers at three-month follow-up visit. Corresponding serum analyte levels were quantified by multiplexed bead array assay as well as ELISA (S100A8/A9, S100A12). Parameters with (A) significant as well as (B) non-significant differences are shown in relation to healthy control levels (range: light grey shading, 95^th^ percentile: broken line). Data are presented as violin plots with individual values and were analyzed by Kruskal Wallis followed by Dunn’s multiple comparison test. *=p<0.05, **=p<0.01, ***=p<0.001. LDA = low disease activity, MDA = moderate disease activity, HDA = high disease activity.

**
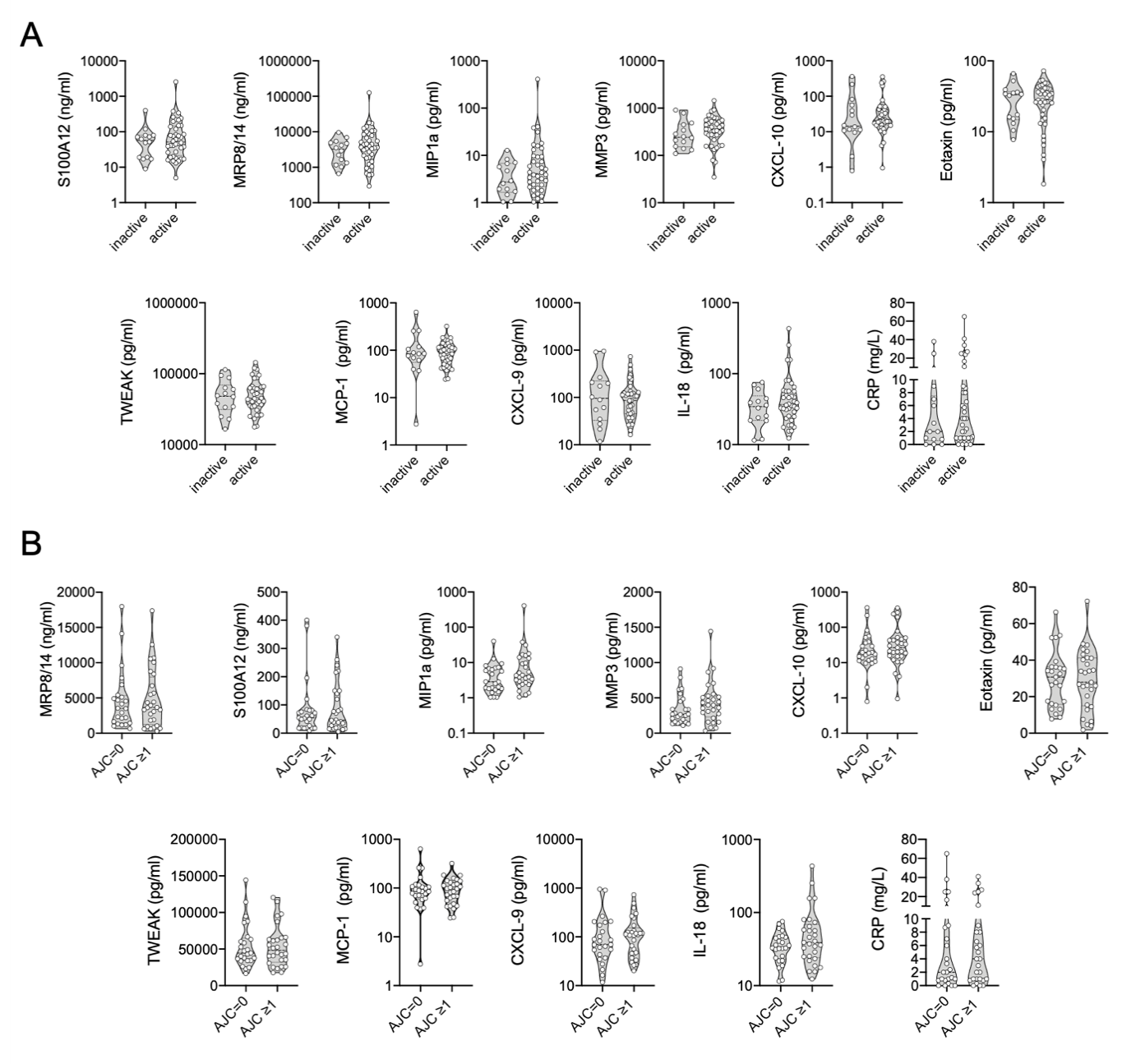
**

Figure S3. Association of baseline serum biomarkers and inflammatory parameters with disease activity outcome at 12 months. (A, B) Inflammatory parameters and serum biomarkers of treatment naïve patients at study inclusion (n=54) were associated with clinical disease activity according to (A) cJADAS (inactive disease: ≤1) or (B) active joint count (AJC) at 12-month follow-up. Indicated parameters assessed at baseline revealed non-significant differences when associated with clinical outcome at 12-month follow-up. Data are presented as violin plots with individual values and were analyzed by Mann-Whitney U test.
